# A study on the mechanism of how sensory impairment affects depression in the elderly: the mediating roles of daily activity capability and social participation

**DOI:** 10.3389/fpsyg.2024.1410422

**Published:** 2024-11-07

**Authors:** Chunjie Huang, Xiaoqing He, Xin Zhang

**Affiliations:** ^1^School of Public Administration, Sichuan University, Chengdu, China; ^2^School of Public Administration, Renmin University of China, Beijing, China

**Keywords:** sensory impairment, depression, daily activity capability, social participation, CHARLS

## Abstract

**Objectives:**

Through a longitudinal study, we explored the relationship between sensory impairments and depression in the elderly, and examined the mediating roles of daily activity capability and social participation within this relationship.

**Methods:**

Based on data from the China Health and Retirement Longitudinal Study (CHARLS) collected in 2015 and 2018, a total of 4,419 individuals aged 60 and above were selected as research participants. Sensory impairments (predictor variables) were assessed in 2015 through self-rated visual and hearing capabilities. Daily activity capability and social participation (mediator variables) were also assessed in 2015, with daily activities assessed using the Basic Activities of Daily Living (BADL) and Instrumental Activities of Daily Living (IADL), and social participation assessed by the quantity of social activity participation. Depression status (outcome variable) was assessed in 2018 using the Center for Epidemiologic Studies Depression Scale (CESD-10). Statistical analysis was conducted using logistic regression and SPSS Macro PROCESS.

**Results:**

First, there is a significant correlation between sensory impairments and an increased risk of depression among the elderly, including visual impairment (VI), hearing impairment (HI), and dual sensory impairment (DSI), all of which increase the likelihood of depression. Second, DSI indirectly affect depression through the cascading mediating effects of daily activity capability and social participation. Finally, in contrast to DSI, when there is only a single sensory impairment, either VI or HI, the cascading mediating effects of daily activity capability and social participation on depression are not statistically significant.

**Conclusion:**

The elderly population with dual sensory impairments requires continued attention to help these individuals adopt preventive measures to halt the onset and worsening of depression.

## Highlights

- Significant Correlation Between Sensory Impairments and Depression: The study finds a strong correlation between sensory impairments, specifically dual sensory impairments, and increased depression risk in the elderly.
- Cascading Mediating Effects of Daily Activity Capability and Social Participation: Dual sensory impairments influence depression indirectly through the combined mediating effects of reduced daily activity capability and lower social participation.
- Distinct Impact of Dual Sensory vs. Single Sensory Impairments: While dual sensory impairments have significant cascading mediating effects on depression, single sensory impairments (either visual or hearing alone) do not show these mediating effects.
- Recommendations for Early Screening and Comprehensive Interventions: The study emphasizes the need for early screening for sensory impairments in the elderly, alongside comprehensive interventions that include both medical support and psychosocial assistance, to enhance their adaptability and quality of life.

## 1 Introduction

Depression, defined as a serious mood disorder, is characterized by an individual’s persistent feelings of sadness, low mood, and a significant decrease in interest or motivation for daily activities (Otte et al., 2016). As a disease that universally affects human mental health, it not only severely impacts the quality of life of the patients themselves (Demyttenaere et al., 2002) but also has profound effects on families and society, including reduced work capacity and strained social relationships (Pratt and Brody, 2008; Koutsimani et al., 2019). Furthermore, depression has become a global public health issue, imposing a significant economic burden (Liu et al., 2020). Data from the World Health Organization show that approximately 4.4% of the global population is affected by depressive disorders with the elderly, especially vulnerable. The prevalence among elderly women aged 55 to 74 exceeds 7.5%, and among elderly men, it is over 5.5% (WHO, 2017). In China, the average depressive symptom score for individuals aged 60 and above is 9.00, with a prevalence rate of 36.25% for depressive disorders (Hu et al., 2018). Major depressive disorder, as the most common type of depression, is characterized by one or more major depressive episodes (Otte et al., 2016). Research targeting the elderly population in China indicates that the point prevalence of major depressive disorder is 2.7%, the 12-month prevalence is 2.3%, and the lifetime prevalence is 2.8% (Wang et al., 2018). Severe depressive states in the elderly are linked to higher medical expenses (Chisholm et al., 2016), a greater likelihood of suicide (Walker et al., 2015), and elevated risks of developing cardiovascular diseases (Hare et al., 2014) and stroke (Dong et al., 2012). Given that China is one of the countries with the fastest aging population globally, with those aged 60 and above expected to account for 28% of the total population by 2040 (WHO, 2022), the issue of depression among the elderly deserves significant attention. Therefore, in the current context of an aging society, a deep understanding of the modifiable risk factors for elderly depression is crucial for more effective prevention and management of the condition.

Depression is a complex mood disorder with multifaceted causes, including biological, psychological, and sociological factors (Engel, 1977). Notably, recent research has identified sensory impairment as a significant risk factor for depression in the elderly (Liu W. et al., 2022). Sensory impairment encompasses the reduction or loss of vision, hearing, touch, taste, and smell, with VI and HI being the most common forms among the elderly (Fischer et al., 2009). Diseases related to VI include age-related macular degeneration (AMD) and cataracts (Varadaraj et al., 2022), whereas HI is usually associated with sensorineural hearing loss (Walling and Dickson, 2012). It is estimated that the prevalence of mild or severe VI among the elderly aged 65 and over ranges from 4 to 12% (Iliffe et al., 2005), while the prevalence of HI among those aged 50 and over is as high as 43% (Feltner et al., 2021). When elderly individuals suffer from both VI and HI, it is referred to as DSI, with its prevalence increasing with age—from 1.5% among those aged 65 to 74 to 10.8% among those aged 85 and older (Rooth, 2017). This phenomenon is particularly significant among the elderly population aged 60 and above in China, with an average of 55% experiencing DSI (Xie et al., 2021). Numerous studies have indicated that sensory impairments negatively affect the mental health of the elderly (Rong et al., 2020), leading to a decline in cognitive abilities (Sense-Cog et al., 2018) and the development of psychiatric disorders such as dementia. Compared to elderly individuals without sensory impairments or with only one type of sensory impairment, those with DSI are at a higher risk of depression (Kiely et al., 2013; Heine and Browning, 2014). Further research has unveiled the neurobiological and socio-psychological impacts that sensory loss might lead to. From a neurobiological perspective, sensory loss can lead to a diminished capacity for the brain to process information, thereby affecting emotional regulation and cognitive functions (Liu C.-J. et al., 2022). Meanwhile, from a socio-psychological viewpoint, the social isolation and loneliness resulting from sensory loss are significant social factors that contribute to the symptoms of depression in the elderly (Harithasan et al., 2020).

In the elderly population, sensory impairments are widely recognized as key factors contributing to decreased daily activity capabilities (Dillon et al., 2010; Heinze et al., 2024). A decline in vision not only limits an individual’s ability to receive visual information but can also lead to a reduction in the capacity to perform everyday life activities, such as increased difficulty reading and decreased mobility autonomy, thereby increasing feelings of loneliness and social isolation (Kempen et al., 2012). Hearing loss restricts the ability of the elderly to communicate, increasing the risk of misunderstandings and communication barriers, leading to decreased social participation and increased loneliness (Ciorba et al., 2012). These impairments not only increase the elderly’s maladaptation to their external environment but can also lead to social isolation, further exacerbating depressive moods. Studies have shown that sensory impairments directly impact the elderly’s ability to complete daily tasks, leading to psychosocial consequences such as depression, anxiety, and societal dissatisfaction (Liljas et al., 2020). Further research has identified decreased daily activity capabilities as an important mediating variable linking sensory impairments to elderly depression (Clark et al., 1999; Brennan et al., 2006). These activities include basic living skills, such as personal hygiene and dressing, as well as more complex activities, such as shopping and using transportation. A decrease in these capabilities can lead to reduced self-efficacy and decreased social participation, both of which are highly correlated with depressive moods (Steffens et al., 1999).

As age increases, sensory functions often decline, directly affecting the elderly’s social activities and their level of social participation (Lövdén et al., 2005; Bourassa et al., 2017). The decline in sensory functions not only directly impacts the individual’s quality of life but may also exacerbate psychological health issues indirectly by limiting social participation (Jaiswal et al., 2020; Levasseur et al., 2022). Social participation can provide emotional support and a sense of belonging, helping to alleviate the feelings of isolation and depressive mood caused by sensory impairments (Hao et al., 2017). Moreover, active forms of social participation, such as engaging in community activities, volunteering, and religious activities, have been shown to have a significant positive impact on the mental health of the elderly (Chiao et al., 2011; Roh et al., 2015). Sensory impairments may also lead to a decrease in information processing capabilities, thereby affecting the elderly’s ability to handle complex information in social interactions. This makes those with lower levels of social participation more prone to feelings of loneliness and social isolation, factors that further increase the risk of depression (Ge et al., 2022). Research further indicates that sensory impairments significantly reduce the elderly’s participation in social activities. This reduction in participation not only decreases their social opportunities but may also lead to an increase in feelings of loneliness and depression (Croezen et al., 2015).

Based on the aforementioned literature, it is evident that previous research has unveiled the relationship between sensory loss and depression in the elderly. However, most studies have concentrated on the direct impact of sensory impairments on depression, overlooking the cascading effects of daily activity capabilities and social participation under the interaction of multiple factors. American psychiatrist George Engel proposed The Biopsychosocial Model in the 1970s (Engel, 1977). This model posits that psychological disorders, including depression, are the result of the combined effects of multiple factors. These include biological factors (such as genetics, changes in brain structure and function), psychological factors (such as personality traits, coping strategies), and social environmental factors (such as social support systems). The sensory impairments and daily activity capabilities mentioned in this paper correspond to biological factors, while social participation corresponds to social environmental factors. Furthermore, The Disablement Process Model describes the process from pathological states to functional limitations and then to disability (Verbrugge and Jette, 1994). Sensory impairments, as initial pathological states, may lead to functional limitations (such as decreased daily activity capability), which in turn lead to disability, restricting an individual’s ability to perform social roles, such as limited social participation. This process of functional decline may increase the risk of depression in the elderly. Therefore, we propose the following hypotheses in this study: (1) There is a correlation between various sensory impairments and depression among the elderly. (2) Daily activity capabilities and social participation will play a cascading mediating role between different sensory impairments and depression.

## 2 Materials and methods

### 2.1 Study population and data source

**Figure 1:**
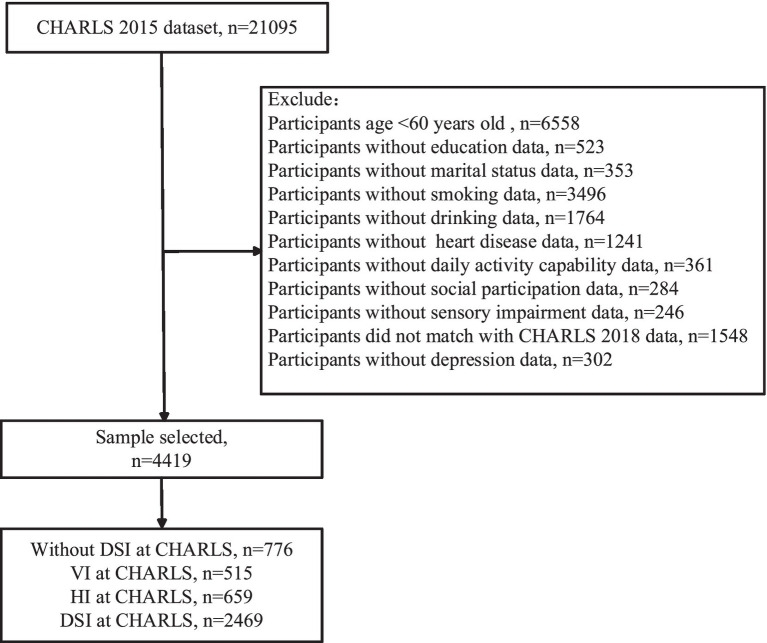
The sample selection in CHARLS.

The data used in this paper originates from the CHARLS, which was initiated by the China Center for Economic Research at Peking University. The purpose of this project is to collect information regarding health and aging among the Chinese population. The sampling scope of the CHARLS project is extensive, covering 150 counties (districts) and 450 villages (communities) across 28 provinces, autonomous regions, and municipalities directly under the Central Government of China, thus effectively representing the condition of the middle-aged and elderly population in China. The CHARLS study has received approval from the Biomedical Ethics Committee of Peking University (IRB00001052-11015). To date, data from five waves in 2011, 2013, 2015, 2018, and 2020 have been accumulated. Considering the potential impact of the COVID-19 pandemic on the mental health of the elderly, this study selected the most recent data from 2015 and 2018 for a longitudinal analysis, excluding data from 2020. Prior to the analysis, we first excluded participants who were under the age of 60 and those lacking crucial variable information. Ultimately, the sample size included in the analysis was 4,419 individuals. The specific sampling process is illustrated in Figure 1.

### 2.2 Sensory impairments

In this study, sensory impairments are categorized into visual impairments and hearing impairments. Through classification of the respondents, we identified four main categories: (1) no sensory impairments, (2) VI only, (3) HI only, and (4) DSI.

VI refers to partial or complete loss of vision. In the CHARLS questionnaire, respondents are asked about their ability to see objects up close and at a distance, with options including Excellent, Very good, Good, Fair, and Poor. Those who choose “Fair” or “Poor” are classified as having a VI, while the others are classified as having no VI. Similarly, HI refers to partial or complete loss of hearing, with the same response options provided in the questionnaire. Respondents who select “Fair” or “Poor” are classified as having a HI, while the others are classified as having no HI. If a respondent has both VI and HI, they are classified as having DSI.

### 2.3 Depressive symptoms

The prevalence of depressive symptoms among the elderly was assessed using a binary variable for depressive symptom risk. Respondents answered questions based on their feelings over the past week, including the following ten items: feeling bothered by little things, difficulty concentrating on work or studies, experiencing low mood, feeling that everything is an effort, having a hopeful outlook on the future, feeling fearful, poor sleep quality, feeling happy, feeling lonely, and feeling unable to carry on with life. These ten items constitute the CESD-10. Each item is scored on a range of 0 to 3: 0 = less than 1 day; 1 = 1 to 2 days; 2 = 3 to 4 days; 3 = 5 to 7 days. Notably, for the statements “I feel hopeful about the future” and “I feel happy,” reverse scoring is used. By summing the scores, we obtain a depression score ranging from 0 to 30. The depressive symptom risk is a binary dependent variable indicating whether the elderly have depressive symptoms (0 = no; 1 = yes), with respondents scoring above 12 considered to have depressive symptoms (Irwin et al., 1999).

### 2.4 Daily activity capability

In this study, “daily activity capability” refers to the ability of the elderly to perform daily living tasks. This concept is divided into two main components: BADL and IADL. Using data from the CHARLS, this study conducted a detailed assessment of these two types of activity capabilities.

BADL involves six fundamental self-care activities: dressing, bathing, eating, getting in and out of bed, using the toilet, and controlling bowel and bladder functions. IADL, on the other hand, includes six more complex household and social activities: doing household chores, shopping, using the telephone, cooking, managing finances, and taking medication.

Respondents selected from four options based on their ability to perform these activities: (1) no difficulty, (2) some difficulty but can do it alone, (3) difficulty and need assistance, (4) unable to do it alone. By summing the scores of these options, a continuous variable ranging from 12 to 48 was derived to quantify the daily activity capability of the elderly. A higher value of this variable indicates greater difficulty encountered in daily activities, that is, poorer daily activity capability.

### 2.5 Social participation

To date, there remains no universally accepted definition of social participation. Some scholars view it as an objective state, namely the number of social contacts or activities a person engages in (Lelkes, 2010). Levasseur et al., in their systematic review of aging literature, defined social participation as “the activity of engaging in actions that provide interaction with others in society or the community (Levasseur et al., 2022).” According to the level of interaction involved, social activities are categorized into six levels: level 1: alone; level 2: in parallel; levels 3 to 6: in interaction. In the questionnaire, respondents were asked whether they had participated in the following activities over the past month: visiting friends and relatives, participating in entertainment activities such as playing mahjong, helping neighbors, participating in dancing or fitness activities, engaging in social organization activities, volunteering or charitable activities, caring for disabled people or patients, attending educational or training courses, trading stocks, surfing the internet, and other social activities. These activities align with Levasseur et al.’s classification. By summing these activities, we derived a continuous variable “social participation” ranging from 0 to 11, where a higher score indicates a greater number of social activities, reflecting the individual’s level of activity and engagement in social interactions.

Furthermore, considering the stricter definition of social participation by other researchers like Shattuck, which is restricted to direct interactions between people (Shattuck et al., 2011), we conducted a robustness test. For this, we excluded internet surfing and stock trading from the calculation of social participation and re-performed the mediation effect test. This helps ensure that our measurement more closely aligns with the core concept of social participation.

### 2.6 Covariates

The covariates in this study include age, biological sex (female, male), educational level (elementary school and below, junior high school, high school, college and above), place of residence (rural, urban), marital status (not married, married), smoking (no, yes), drinking (no, yes), and heart disease (no, yes). These variables are included to account for potential confounding factors that may influence the relationship between sensory impairments, depressive symptoms, daily activity capability, and social participation among the elderly.

### 2.7 Statistical analysis

In the descriptive statistical analysis, continuous variables are presented using mean ± standard deviation, while categorical variables are shown using counts and percentages. To explore the differences between each subgroup and the group without sensory impairments, we used the t-test. Additionally, we conducted Kruskal-Wallis and *Post hoc* tests to further analyze differences among the VI, HI, and DSI groups. In the regression analysis, we employed logistic regression where sensory impairments assessed in 2015 served as the predictor variable, and depression assessed in 2018 was the outcome variable. We set up dummy variables, using the group without sensory impairments as the reference group. For the analysis of cascading mediating effects, we utilized Model 6 from the PROCESS macro developed by Hayes in SPSS. Specifically, we created three sample subsets to explore the mediating effects. The first subset included only individuals without sensory impairments and those with DSI, the second subset included only those without sensory impairments and those with VI, and the third subset included only those without sensory impairments and those with HI. That is, the predictor variables in all three mediation models were binary. Specifically, the predictor variables in the three models were DSI, VI, and HI, respectively, all assessed in 2015. The mediating variables, social participation and daily activity capabilities, were also assessed in 2015. The outcome variable, depression in the elderly, was assessed in 2018. In this article, a *p*-value <0.05 is considered statistically significant.

To verify the robustness of our findings, we conducted several sensitivity analyses. First, we used multiple imputation to fill in missing values for covariates, and repeated all analyses post-imputation. Second, for social participation, we applied a stricter definition, excluding internet usage and stock trading activities, and then re-conducted the cascading mediation effect analysis.

## 3 Results

### 3.1 Sample characteristics and differences between subgroups

Table 1 presents the sample characteristics and differences between subgroups. It reports the distribution and mean values (including standard deviations) for each variable, as well as the statistical significance of differences between each subgroup and the group without sensory impairments (*p*-values). Among the total of 4,419 respondents, 515 reported having VI, 659 had HI, and 2,469 suffered from DSI, totaling 3,643 individuals affected by some form of sensory impairment. This highlights the prevalence of sensory impairments among the elderly. The analysis of differences between groups shows that compared to the group without sensory impairments, members of the VI group are more prone to depression and less likely to smoke; the HI group has a higher probability of depression, is more likely to be male, and has poorer daily activity capabilities; the DSI group has a greater likelihood of depression, tends to be older, has lower educational levels, lives in rural areas, and exhibits lower daily activity capabilities and social participation. Further analysis of differences between groups is detailed in Supplementary Table S1. Among all participants with sensory impairments, the DSI group demonstrates the lowest daily activity capability and the least participation in social activities.

**Table 1:** Sample characteristics and differences between subgroups, grouped by sensory impairment.

	Without DSI	VI	*p*	HI	*p*	DSI	*p*	Total
	(*N* = 776)	(*N* = 515)		(*N* = 659)		(*N* = 2,469)		(*N* = 4,419)
Depression			0.005		0.005		<0.001	
No	604 (77.8%)	365 (70.9%)		470 (71.3%)		1,538 (62.3%)		2,977 (67.4%)
Yes	172 (22.2%)	150 (29.1%)		189 (28.7%)		931 (37.7%)		1,442 (32.6%)
Age			0.779		0.166		0.042	
Mean (SD)	66.52 (7.30)	66.40 (7.57)		67.07 (7.52)		67.15 (7.51)		66.94 (7.49)
Biological sex			0.08		0.025		0.772	
Female	644 (83.0%)	446 (86.6%)		516 (78.3%)		2060 (83.4%)		3,666 (83.0%)
Male	132 (17.0%)	69 (13.4%)		143 (21.7%)		409 (16.6%)		753 (17.0%)
Education			0.783		0.127		<0.001	
Elementary school and below	578 (74.5%)	385 (74.8%)		508 (77.1%)		2049 (83.0%)		3,520 (79.7%)
Junior high school	110 (14.2%)	71 (13.8%)		91 (13.8%)		271 (11.0%)		543 (12.3%)
High school	76 (9.8%)	56 (10.9%)		55 (8.3%)		128 (5.2%)		315 (7.1%)
College and above	12 (1.5%)	3 (0.6%)		5 (0.8%)		21 (0.9%)		41 (0.9%)
Living area			0.88		0.827		<0.001	
Rural	547 (70.5%)	361 (70.1%)		468 (71.0%)		1917 (77.6%)		3,293 (74.5%)
Urban	229 (29.5%)	154 (29.9%)		191 (29.0%)		552 (22.4%)		1,126 (25.5%)
Marital status			0.072		0.477		0.972	
Not married	206 (26.5%)	114 (22.1%)		186 (28.2%)		657 (26.6%)		1,163 (26.3%)
Married	570 (73.5%)	401 (77.9%)		473 (71.8%)		1812 (73.4%)		3,256 (73.7%)
Smoking			0.002		0.804		0.208	
No	718 (92.5%)	498 (96.7%)		612 (92.9%)		2,316 (93.8%)		4,144 (93.8%)
Yes	58 (7.5%)	17 (3.3%)		47 (7.1%)		153 (6.2%)		275 (6.2%)
Drinking			0.056		0.826		0.213	
No	623 (80.3%)	435 (84.5%)		526 (79.8%)		2,031 (82.3%)		3,615 (81.8%)
Yes	153 (19.7%)	80 (15.5%)		133 (20.2%)		438 (17.7%)		804 (18.2%)
Heart disease			0.096		0.52		0.585	
No	684 (88.1%)	469 (91.1%)		588 (89.2%)		2,158 (87.4%)		3,899 (88.2%)
Yes	92 (11.9%)	46 (8.9%)		71 (10.8%)		311 (12.6%)		520 (11.8%)
Daily activity capability			0.234		0.011		<0.001	
Mean (SD)	13.87 (3.60)	14.13 (3.97)		14.39 (4.13)		15.22 (4.79)		14.73 (4.45)
Social participation			0.901		0.134		<0.001	
Mean (SD)	0.89 (1.13)	0.89 (1.09)		0.80 (0.97)		0.67 (0.95)		0.75 (1.01)

### 3.2 Regression analysis

Table 2 displays the association between sensory impairment and depression. Model 1 shows that there are significant associations between sensory impairment and depression. VI was associated with increase in depression scores (OR = 1.443, CI = [1.119, 1.862], *p* = 0.005), indicating a statistically significant impact on depressive symptoms. HI also demonstrated a significant association with increased depression (OR = 1.412, CI = [1.112, 1.794], *p* = 0.005). DSI showed a significant association with depression (OR = 2.126, CI = [1.762, 2.565], *p* < 0.001). These findings suggest that individuals with sensory impairments, including those with VI or HI, or both, may be at risk for depression.

**Table 2:** The association between sensory impairment and depression.

	Model 1			Model 2		
	OR	95% CI	*p*	OR.	95% CI	*p*
VI	1.443**	(1.119–1.862)	0.005	1.483**	(1.145–1.922)	0.003
HI	1.412**	(1.112–1.794)	0.005	1.451**	(1.137–1.850)	0.003
DSI	2.126***	(1.762–2.565)	<0.001	2.056***	(1.698–2.489)	<0.001
Age				0.979***	(0.970–0.988)	<0.001
Biological sex				0.628***	(0.504–0.784)	<0.001
Education				0.723***	(0.636–0.823)	<0.001
Living area				0.644***	(0.545–0.762)	<0.001
Marital status				0.750***	(0.645–0.871)	<0.001
Smoking				1.369	(0.996–1.881)	0.053
Drinking				0.884	(0.735–1.061)	0.186
Heart disease				1.469***	(1.208–1.786)	<0.001
Constant	0.285***	(0.240–0.337)	<0.001	2.466*	(1.204–5.049)	0.014
*N*	4,419			4,419		

Data based on listwise deletion. Logistic regression analysis was used, with sensory impairments as the predictor variables and depression as the outcome variable. *p ≤ 0.05, **p ≤ 0.01, ***p ≤ 0.001.

In the analysis of Model 2, we controlled for demographic variables and health factor variables to ensure the robustness of our findings regarding the association between sensory impairments and depression. Specifically, we included age, biological sex, education, residential area, marital status, smoking and drinking habits, and heart disease as covariates. The results showed that the OR values did not change significantly, and the groups with VI (OR = 1.483, CI = [1.145, 1.922], *p* = 0.003), HI (OR = 1.451, CI = [1.137, 1.850], *p* = 0.003), and DSI (OR = 2.056, CI = [1.698, 2.489], *p* < 0.001) were still significantly correlated with depression. Furthermore, Age (OR = 0.979, CI = [0.970, 0.988], *p* < 0.001), Biological Sex (OR = 0.628, CI = [0.504, 0.784], *p* < 0.001), Education (OR = 0.723, CI = [0.636, 0.823], *p* < 0.001), Living Area (OR = 0.644, CI = [0.545, 0.762], *p* < 0.001), Marital Status (OR = 0.750, CI = [0.645, 0.871], *p* < 0.001) were negatively correlated with depression in the elderly, while Heart Disease (OR = 1.469, CI = [1.208, 1.786], *p* < 0.001) was positively correlated with depression in the elderly.

### 3.3 Cascading mediation effect test

**Figure 2:**
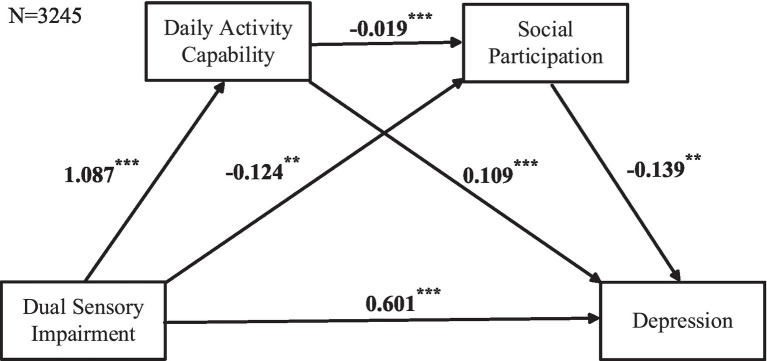
Cascading mediation model including the no sensory impairments and DSI groups. Data based on listwise deletion. Cascading mediation regression analysis was used, with DSI as the predictor variable, daily activity capabilities and social participation as mediating variables, and depression as the outcome variable.

Figure 2 shows that the independent variable is DSI, with daily activity capability and social participation as mediating variables, and depression as the outcome variable. The cascading mediation model analysis indicates that first, DSI has a direct positive effect on depression (*β* = 0.601, *p* < 0.001), while significantly enhancing daily activity capability (β = 1.087, *p* < 0.001). Moreover, daily activity capability has a significant positive effect on depression (β = 0.109 *p* < 0.001). Furthermore, DSI negatively predicts social participation (β = −0.124, *p* < 0.05), and social participation significantly alleviates symptoms of depression (β = −0.139, *p* < 0.05). Most importantly, daily activity capability has a negative effect on social participation (β = −0.019, *p* < 0.001).

Table 3 presents more detailed test results. We found that the direct effect of DSI on depression is significant, with an effect size of 0.6018. Its indirect effect size is 0.1388, and its 95% confidence interval does not include 0, further confirming the significance of the indirect effect. In addition, daily activity capability and social participation, as mediating variables, together explain 18.74% of the total effect. Specifically, the mediating effect can be divided into three paths: (1) DSI indirectly affects depression by affecting daily activity capability, this path’s mediating effect is significant, explaining 16.01% of the total effect; (2) DSI indirectly affects depression by affecting social participation, this path’s mediating effect is also significant, explaining 2.34% of the total effect; (3) DSI affects depression through the cascading mediating path of daily activity capability and social participation, this path’s mediating effect is significant, explaining 0.39% of the total effect. These results indicate that the cascading mediating role is established.

**Table 3:** The cascading mediating effect.

Model pathways	Effect	Boot SE	95% CI		Relative mediation effect %
			Lower	Upper	
Including the no sensory impairments and DSI groups					
Direct effect	0.602	0.100	0.450	0.798	81.242
DSI → Daily Activity Capability→Depression	0.119	0.020	0.082	0.161	16.059
DSI → Social Participation→Depression	0.017	0.008	0.004	0.036	2.294
DSI → Daily Activity Capability→Social Participation→Depression	0.003	0.001	0.001	0.005	0.405
Total mediation effect	0.139	0.022	0.099	0.185	18.758
Including the no sensory impairments and VI groups					
Direct effect	0.379	0.138	0.109	0.649	–
VI → Daily Activity Capability → Depression	0.045	0.030	0.009	0.110	–
VI → Social Participation → Depression	−0.002	0.008	−0.020	0.013	–
VI → Daily Activity Capability → Social Participation → Depression	0.001	0.001	−0.004	0.004	–
Total mediation effect	0.044	0.032	−0.016	0.112	–
Including the no sensory impairments and HI groups					
Direct effect	0.352	0.127	0.102	0.602	–
HI → Daily Activity Capability → Depression	0.038	0.018	0.005	0.076	–
HI → Social Participation → Depression	0.004	0.007	−0.008	0.021	–
HI → Daily Activity Capability → Social Participation → Depression	0.001	0.001	−0.004	0.004	–
Total mediation effect	0.043	0.020	0.007	0.084	–

**Figure 3:**
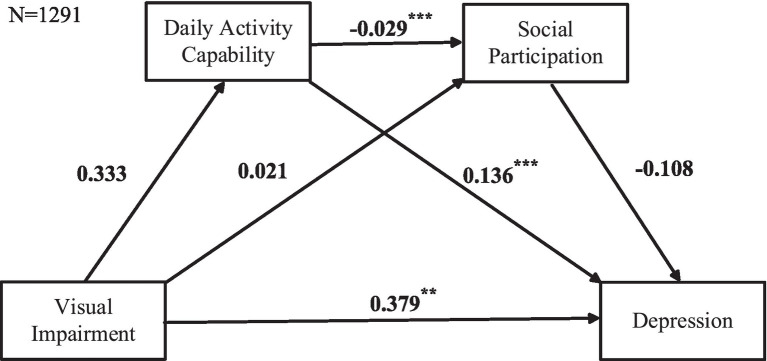
Cascading mediation model including the no sensory impairments and VI groups. Data based on listwise deletion. Cascading mediation regression analysis was used, with VI as the predictor variable, daily activity capabilities and social participation as mediating variables, and depression as the outcome variable.

In Figure 3, the independent variable is VI, the mediating variables are Daily Activity Capabilities and Social Participation, and the outcome variable is Depression. The cascading mediation model analysis shows that VI has no significant effect on Daily Activity Capabilities or Social Participation, and Social Participation has no significant effect on Depression. Further analysis in Table 3 confirms that two pathways are not valid: VI → Social Participation→Depression (95%CI = [−0.0197, 0.0127]) and VI → Daily Activity Capability→Social Participation→Depression (95%CI = [−0.0004, 0.0038]). Therefore, the cascading mediation model among VI, Daily Activity Capabilities, Social Participation, and Depression is not valid.

**Figure 4:**
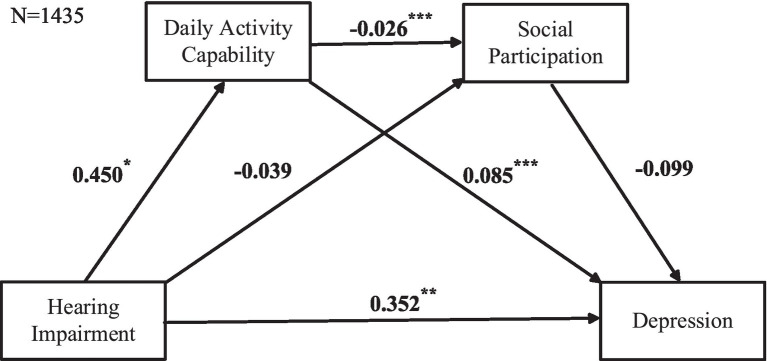
Cascading mediation model including the no sensory impairments and HI groups. Data based on listwise deletion. Cascading mediation regression analysis was used, with HI as the predictor variable, daily activity capabilities and social participation as mediating variables, and depression as the outcome variable.

In Figure 4, the independent variable is HI, the mediating variables are Daily Activity Capabilities and Social Participation, and the outcome variable is Depression. The cascading mediation model analysis shows that HI has no significant effect on Social Participation, and Social Participation has no significant effect on Depression. Further analysis in Table 3 confirms that two pathways are not valid: HI → Social Participation→Depression (95%CI = [−0.0076, 0.0208]) and HI → Daily Activity Capability→Social Participation→Depression (95%CI = [−0.0004, 0.0038]). Therefore, the cascading mediation model among HI, Daily Activity Capabilities, Social Participation, and Depression is not valid.

### 3.4 Sensitivity analysis

Sensitivity analysis indicates that the results remain unchanged even after using multiple imputation methods to fill in missing values (see Supplementary Tables S2, S3; Supplementary Figures S1–S3). Furthermore, we applied a stricter definition of social participation by excluding internet surfing and stock trading activities. We then reanalyzed the cascading mediation effects based on the original data using listwise deletion (see Supplementary Figures S4–S6; Supplementary Table S4). The conclusions from the earlier mediation tests still hold.

## 4 Discussion

Based on the longitudinal data from the 2015 and 2018 CHARLS, we tested the relationship between sensory impairments and depression in the elderly and investigated the cascading mediating roles of daily activity capability and social participation within this relationship. Our findings are as follows: First, there is a significant correlation between sensory impairments—including VI, HI, and DSI—and an increased risk of depression among the elderly. Second, DSI indirectly affect depression through the cascading mediating effects of daily activity capability and social participation. Finally, in contrast to DSI, when there is only a single sensory impairment, either VI or HI, the cascading mediating effects of daily activity capability and social participation on depression are not statistically significant.

Our research findings indicate that VI, HI, and DSI all significantly exacerbate depressive symptoms in the elderly, consistent with previous research findings (Rong et al., 2020). Specifically, vision loss is considered a consistent predictor of both the onset and persistence of depression among the elderly (Chou, 2008). Among those with impaired vision, 13.5% suffer from depression, compared to 4.6% of those with good vision (Evans et al., 2007). Furthermore, VI are associated with the emergence of depressive symptoms within 5 years, with 27% of cases identified with VI 5 years prior reporting symptoms of depression, compared to 10.8–11.5% in the control group (Hong et al., 2015). Turning to HI, numerous studies have also found that auditory issues significantly impact depressive outcomes. A study highlights that the psychological state and mental functions of elderly participants using hearing aids significantly improved, suggesting that adapting to hearing aids may be a viable solution to combat depression in this population (Acar et al., 2011). Additionally, research shows that HI increase the risk of depression, regardless of demographic factors and medical history, with severe HI significantly raising the risk of depression across all age groups (Kim et al., 2017). Beyond the impact of single sensory loss, concurrent vision and hearing problems impose an additional burden on mental health (Kiely et al., 2013).

Our study results further explain the mechanism by which DSI lead to depression among the elderly. Firstly, DSI reduce the elderly’s daily activity capability, subsequently leading to depression. This mechanism is in line with the Self-Determination Theory (SDT) proposed by Deci and Ryan, which suggests that when the needs for autonomy, competence, and relatedness are challenged or unmet, individuals may experience negative emotions such as depression and anxiety (Deci and Ryan, 2013). DSI restrict their ability to make autonomous decisions and independently perform daily activities, directly impacting their need for autonomy, which could lead to the emergence or exacerbation of depressive symptoms (Han et al., 2019). Further research shows that elderly individuals with self-reported DSI exhibited higher levels of functional impairment both at the initial assessment and during follow-ups over the next 2 years (Brennan et al., 2006). A longitudinal study on frail Dutch individuals aged 60 and above found that compared to those without sensory loss or with only one sensory impairment, individuals with DSI faced more restrictions in IADL (Mueller-Schotte et al., 2019). These restrictions in activities are significantly negatively correlated with symptoms of depression (Mohamadzadeh et al., 2020). Secondly, DSI reduce the elderly’s social participation, subsequently leading to depression (Pancani et al., 2021). This may be because sensory impairments not only limit an individual’s social activities, reducing communication with the outside world, but also increase dependence on external assistance, thereby causing a decrease in self-esteem and an increase in feelings of loneliness. This aligns with the Social Connection Theory, which emphasizes the importance of social connections for maintaining psychological health (Cacioppo and Patrick, 2008). Elderly individuals with DSI feel lonely due to limited participation in social activities outside the family (Crowe et al., 2020). The communication barriers caused by DSI further exacerbate this phenomenon (Resnick et al., 1997; Heine and Browning, 2014). Lastly, daily activity capability and social participation play a cascading mediating role in the relationship between sensory impairments and depression. The biopsychosocial model indicates that the onset mechanism of depression results from the interplay of multiple factors (Engel, 1977). Specifically, functional limitations complicate and hinder social interactions. Elderly individuals may avoid socializing as much as possible, leading to a reduction in social support and an increase in feelings of isolation and exclusion. Research has demonstrated that social networks can act as a buffer, mitigating the negative impact of decreased daily living activity capability on depression (Guo et al., 2022).

In the examination of cascading mediating effects, we found that, in contrast to DSI, when there is only a single sensory impairment, either VI or HI, the cascading mediating effects of daily activity capability and social participation on depression are not statistically significant. The reason for this phenomenon can be explained by the Sensory Compensation Theory, which posits that when one sensory function is impaired, individuals may develop increased sensitivity or functionality in other senses to compensate for the lost sense (Bach-y-Rita and Kercel, 2003). For individuals with only a single sensory impairment, visual and auditory functions can compensate for each other (Bolognini et al., 2012). This compensatory ability can help them perform better in daily activities and mitigate barriers to social participation. Supplementary Table S1 shows that, compared to individuals with DSI, those with only VI or only HI demonstrate stronger daily activity capabilities and greater social participation; however, there are no significant differences between VI and HI individuals in these aspects. These findings somewhat confirm our hypothesis.

From a theoretical standpoint, our study deepens the understanding of how sensory impairments affect the mechanisms underlying depression in the elderly. By verifying the cascading mediating effects of daily activity capability and social participation between DSI and depression, we support the applicability of the Biopsychosocial model and the Disablement Process model in explaining this process. From a practical perspective, our findings offer new directions for interventions aimed at preventing depression in the elderly. For elderly individuals with DSI, emphasis should be placed on improving their daily activity capabilities and promoting social participation. Specific measures include providing sensory rehabilitation training, equipping assistive devices to enhance sensory functions, and designing social activities suitable for their abilities to boost social interaction. For those with single sensory impairments, preventive efforts should be made to avert progression to DSI. Policymakers and healthcare providers should comprehensively consider the multiple impacts of sensory impairments on the mental health of the elderly. Developing integrated intervention measures that not only address the loss of sensory functions but also enhance daily functioning and social participation can effectively prevent the onset of depression.

This study has several limitations. First, the assessment of sensory impairments in the elderly is based on self-report rather than medical diagnosis, which may threaten the accuracy of the impairment evaluation. Second, the measurement of social participation is based on the number of social activities the elderly engage in, but according to Boutot and Bryant’s definition of social participation, subjective experiences such as preferences are also an important component (Boutot and Bryant, 2005). Therefore, measuring by the number of activities may not fully reflect the extent of social participation among the elderly. Third, this paper is based on data from China, which may have regional limitations, meaning that these findings should be cautiously generalized to other countries or cultures. Therefore, it is suggested that a comprehensive study based on multinational data be conducted to validate the universality of these results.

## Data Availability

Publicly available datasets were analyzed in this study. This data can be found here: https://charls.pku.edu.cn/en/.
