# Supplementary material for: A study on the mechanism of how sensory impairment affects depression in the elderly: the mediating roles of daily activity capability and social participation
Source: Front Psychol. 2024 Nov 7;15:1410422. doi: 10.3389/fpsyg.2024.1410422 (PMC11578716; doi:10.3389/fpsyg.2024.1410422)
Supplement: Supplementary file 1 [file Table_1.DOCX]

**supplementary material**

**Supplementary Table 1. Summary of** **Kruskal-Wallis and Post Hoc Tests for VI, HI, and DSI Groups**

|  | Kruskal-Wallis test: χ^2^ | p-Value | VI vs HI:  p-Value | VI vs DSI:  p-Value | HI vs DSI:  p-Value |
| --- | --- | --- | --- | --- | --- |
| Depression | 27.349 | <0.001 | 0.448 | 0.001 | <0.001 |
| Daily Activity Capability | 45.648 | <0.001 | 0.176 | <0.001 | <0.001 |
| Social Participation | 27.852 | <0.001 | 0.190 | <0.001 | <0.001 |
| N | 4419 |  | 1174 | 2984 | 3128 |

**Supplementary Table 2. The association between sensory impairment and depression**

|  | Model 1 |  |  | Model 2 |  |  |
| --- | --- | --- | --- | --- | --- | --- |
|  | OR | 95% CI | p | OR | 95% CI | p |
| VI | 1.576*** | (1.299 - 1.912) | <0.001 | 1.553*** | (1.276 - 1.890) | <0.001 |
| HI | 1.378*** | (1.154 - 1.644) | <0.001 | 1.439*** | (1.202 - 1.723) | <0.001 |
| DSI | 2.254*** | (1.954 - 2.601) | <0.001 | 2.197*** | (1.899 - 2.542) | <0.001 |
| Age |  |  |  | 0.981*** | (0.974 - 0.987) | <0.001 |
| Biological Sex |  |  |  | 0.659*** | (0.588 - 0.739) | <0.001 |
| Education |  |  |  | 0.757*** | (0.696 - 0.823) | <0.001 |
| Living Area |  |  |  | 0.691*** | (0.613 - 0.778) | <0.001 |
| Marital Status |  |  |  | 0.746*** | (0.668 - 0.834) | <0.001 |
| Smoking |  |  |  | 1.062 | (0.906 - 1.244) | 0.458 |
| Drinking |  |  |  | 0.828** | (0.736 - 0.931) | <0.001 |
| Heart Disease |  |  |  | 1.323*** | (1.145 - 1.529) | <0.001 |
| Constant | 0.251*** | (0.220 - 0.286) | <0.001 | 2.228** | (1.332 - 3.727) | 0.002 |
| N | 8,770 |  |  | 8,770 |  |  |

*Notes:* Data based on multiple imputation. Logistic regression analysis was used, with sensory impairments as the predictor variables and depression as the outcome variable.

**Supplementary Table 3. The cascading mediating effect**

| Model Pathways | Effect | Boot SE | 95% CI | | Relative Mediation Effect % |
| --- | --- | --- | --- | --- | --- |
|  |  |  | Lower | Upper |  |
| **Including the No Sensory Impairments and DSI Groups** |  |  |  |  |  |
| Direct effect | 0.676 | 0.076 | 0.527 | 0.825 | 84.080% |
| DSI→Daily Activity Capability→Depression | 0.114 | 0.014 | 0.086 | 0.143 | 14.179% |
| DSI→Social Participation→Depression | 0.013 | 0.006 | 0.003 | 0.025 | 1.617% |
| DSI→Daily Activity Capability→Social Participation→Depression | 0.002 | 0.001 | 0.001 | 0.004 | 0.249% |
| Total mediation effect | 0.128 | 0.016 | 0.0990 | 0.160 | 15.920% |
| **Including the No Sensory Impairments and VI Groups** |  |  |  |  |  |
| Direct effect | 0.388 | 0.103 | 0.186 | 0.591 | —— |
| VI→Daily Activity Capability→Depression | 0.066 | 0.023 | 0.026 | 0.114 | —— |
| VI→Social Participation→Depression | 0.005 | 0.006 | -0.003 | 0.018 | —— |
| VI→Daily Activity Capability→Social Participation→Depression | 0.002 | 0.001 | -0.002 | 0.005 | —— |
| Total mediation effect | 0.073 | 0.024 | 0.031 | 0.123 | —— |
| **Including the No Sensory Impairments and HI Groups** |  |  |  |  |  |
| Direct effect | 0.339 | 0.094 | 0.155 | 0.523 | —— |
| HI→Daily Activity Capability→Depression | 0.030 | 0.012 | 0.009 | 0.055 | —— |
| HI→Social Participation→Depression | 0.003 | 0.004 | -0.004 | 0.013 | —— |
| HI→Daily Activity Capability→Social Participation→Depression | 0.001 | 0.001 | -0.001 | 0.003 | —— |
| Total mediation effect | 0.034 | 0.013 | 0.011 | 0.061 | —— |

**Supplementary Table 4. The cascading mediating effect**

| Model Pathways | Effect | Boot SE | 95% CI | | Relative Mediation Effect % |
| --- | --- | --- | --- | --- | --- |
|  |  |  | Lower | Upper |  |
| **Including the No Sensory Impairments and DSI Groups** |  |  |  |  |  |
| Direct effect | 0.606 | 0.100 | 0.410 | 0.802 | 82.114% |
| DSI→Daily Activity Capability→Depression | 0.119 | 0.020 | 0.082 | 0.161 | 16.125% |
| DSI→Social Participation→Depression | 0.010 | 0.006 | 0.004 | 0.025 | 1.355% |
| DSI→Daily Activity Capability→Social Participation→Depression | 0.003 | 0.001 | 0.001 | 0.005 | 0.407% |
| Total mediation effect | 0.132 | 0.021 | 0.093 | 0.176 | 17.886% |
| **Including the No Sensory Impairments and VI Groups** |  |  |  |  |  |
| Direct effect | 0.380 | 0.138 | 0.110 | 0.650 | —— |
| VI→Daily Activity Capability→Depression | 0.045 | 0.030 | -0.010 | 0.106 | —— |
| VI→Social Participation→Depression | -0.004 | 0.008 | -0.023 | 0.008 | —— |
| VI→Daily Activity Capability→Social Participation→Depression | 0.001 | 0.001 | -0.001 | 0.003 | —— |
| Total mediation effect | 0.042 | 0.032 | -0.018 | 0.107 | —— |
| **Including the No Sensory Impairments and HI Groups** |  |  |  |  |  |
| Direct effect | 0.353 | 0.127 | 0.103 | 0.602 | —— |
| HI→Daily Activity Capability→Depression | 0.038 | 0.018 | 0.006 | 0.076 | —— |
| HI→Social Participation→Depression | 0.001 | 0.006 | -0.009 | 0.015 | —— |
| HI→Daily Activity Capability→Social Participation→Depression | 0.001 | 0.001 | -0.001 | 0.003 | —— |
| Total mediation effect | 0.041 | 0.019 | 0.006 | 0.080 | —— |

**Supplementary Figure 1. Cascading Mediation Model Including the No Sensory Impairments and DSI Groups**

*Notes:* Data based on multiple imputation. Cascading mediation regression analysis was used, with DSI as the predictor variable, daily activity capabilities and social participation as mediating variables, and depression as the outcome variable.

**Supplementary Figure 2. Cascading Mediation Model Including the No Sensory Impairments and VI Groups**

*Notes:* Data based on multiple imputation. Cascading mediation regression analysis was used, with VI as the predictor variable, daily activity capabilities and social participation as mediating variables, and depression as the outcome variable.

**Supplementary Figure 3. Cascading Mediation Model Including the No Sensory Impairments and HI Groups**

*Notes:* Data based on multiple imputation. Cascading mediation regression analysis was used, with HI as the predictor variable, daily activity capabilities and social participation as mediating variables, and depression as the outcome variable.

**Supplementary Figure 4. Cascading Mediation Model Including the No Sensory Impairments and DSI Groups**

*Notes:* Data based on listwise deletion. A stricter definition of social participation was applied. Cascading mediation regression analysis was used, with DSI as the predictor variable, daily activity capabilities and social participation as mediating variables, and depression as the outcome variable.

**Supplementary Figure 5. Cascading Mediation Model Including the No Sensory Impairments and VI Groups**

*Notes:* Data based on listwise deletion. A stricter definition of social participation was applied. Cascading mediation regression analysis was used, with VI as the predictor variable, daily activity capabilities and social participation as mediating variables, and depression as the outcome variable.

**Supplementary Figure 6. Cascading Mediation Model Including the No Sensory Impairments and HI Groups**

*Notes:* Data based on listwise deletion. A stricter definition of social participation was applied. Cascading mediation regression analysis was used, with HI as the predictor variable, daily activity capabilities and social participation as mediating variables, and depression as the outcome variable.
